# Back from the brink: a mesenchymal stem cell infusion rescues kidney function in acute experimental rhabdomyolysis

**DOI:** 10.1186/scrt497

**Published:** 2014-09-11

**Authors:** Michelle M Duffy, Matthew D Griffin

**Affiliations:** Orbsen Therapeutics Ltd, National University of Ireland, Orbsen Building, University Road, Galway, Ireland; Regenerative Medicine Institute (REMEDI) and College of Medicine, Nursing and Health Sciences, National University of Ireland Galway, REMEDI, Biosciences, Corrib Village, Dangan, Galway, Ireland

## Abstract

Systemic administration of mesenchymal stem (stromal) cells (MSCs) has shown benefit in a range of experimental models of acute kidney injury, although the reported mechanisms of action and requirement for MSC localization to the kidney have varied. Geng and colleagues now demonstrate that a single intravenous infusion of MSCs given 6 hours after induction of acute muscle necrosis (rhabdomyolysis) robustly ameliorates the resulting acute kidney injury and promotes early intra-renal accumulation of CD206^+^ (M2) macrophages. The benefit occurred in the absence of MSC localization to the kidney and could be reproduced by adoptive transfer of *ex vivo*-programmed M2 macrophages.

## Introduction

In a recent issue of *Stem Cell Research & Therapy*, Geng and colleagues [1] reported that a single intravenous infusion of autologous mesenchymal stem (stromal) cells (MSCs) given 6 hours after induction of acute muscle necrosis (rhabdomyolysis) in mice robustly ameliorates the resulting acute kidney injury (AKI). Improvements in kidney function and structure and epithelial cell proliferation were associated with early intra-renal accumulation of CD206^+^ (M2) macrophages and were reversed when phagocytic cells were eliminated by liposomal clodronate 24 hours after the onset of rhabdomyolysis. Interestingly, adoptive transfer of mouse macrophages (RAW 264.7 cells) co-cultured with MSCs produced a similar beneficial effect [1]. Thus, the study provides clear evidence that MSC administration shortly after induction of rhabdomyolysis can mediate a strong tissue-protective effect via re-programming (or ‘alternative activation’) of macrophages or their immediate precursor monocytes or both.

## Mechanisms of action of mesenchymal stem cells in rhabdomyolysis-associated acute kidney injury

Rhabdomyolysis is frequently associated with AKI as a result of the release of myoglobin breakdown products (in particular, ferrihemate) into the bloodstream following the disintegration of muscle fibers. Individuals may be genetically predisposed to or may acquire rhabdomyolysis as a result of trauma, drug toxicity, immobilization, or epileptic seizures [2]. Progression to myoglobinuric AKI occurs in 8% to 20% of patients with rhabdomyolysis [3] while the mortality rate increases from 8% to 10% to as high as 50% with development of AKI [2]. A key strength of the study by Geng and colleagues in regard to future clinical translation is that the timing of MSC administration (6 hours after intramuscular glycerol injection) is in keeping with their use by front-line emergency medicine personnel in victims of trauma or acute illness with evidence of severe muscle injury.

MSCs are immune-modulatory, plastic-adherent cells with the capacity to differentiate into chondrocytes, osteoblasts, and adipocytes under controlled culture conditions. In the bone marrow, they constitute part of the perivascular hematopoietic niche, but they are also present in a wide range of other adult tissues. MSCs have been reported to protect against AKI in multiple pre-clinical models, including AKI associated with glycerol-induced rhabdomyolysis in rodents [1, 4–6]. The various proposed mechanisms for MSC benefits in AKI are discussed in detail in a recent review [7], but it is worth noting that the intrinsic reparative properties of MSCs are more frequently ascribed to their paracrine (‘trophic’) effects than to their ability to trans-differentiate into functional renal parenchymal cells. In this regard, an important experimental finding from the study by Geng and colleagues is that MSCs did not demonstrably migrate to the injured kidney within the first 24 hours of induction of rhabdomyolysis but were detected in damaged muscle and in the lungs [1]. This observation is in keeping with an extra-renal primary site of action of infused MSCs from which reno-protective effects are mediated by induced soluble factors (for example, IL-10) and migratory cell populations (for example, re-programmed monocyte/macrophages) [8]. Notably, however, previous studies by Herrera and colleagues [5, 6] in which MSCs were administered intravenously to mice with glycerol-induced rhabdomyolysis demonstrated CD44/hyaluronic acid-dependent migration of MSCs to injured kidney that was associated with enhanced renal repair and with MSC engraftment into tubular epithelium, interstitium, and peritubular capillaries. In these studies, MSCs were injected at a later time-point (3 days post-glycerol) than in the study by Geng and colleagues, suggesting that timing of administration and perhaps other factors such as culture conditions, passage number, and surface receptor profile may significantly influence *in vivo* mechanism of action.

Geng and colleagues observed that serum concentrations of IL-10 were increased but that IL-6 and tumor necrosis factor-alpha were decreased in MSC-treated mice [1]. Consistent with this shift in archetypal pro- and anti-inflammatory cytokines, the authors detected enhanced M2 polarization in the presence of MSCs on the basis of expression of CD206/mannose receptor. These findings concur with those of Németh and colleagues [8], who demonstrated that increased expression of IL-10 by MSC-educated macrophages was responsible for enhanced survival in a sepsis model. In that study, MSC-derived prostaglandin E_2_ (PGE_2_) altered the phenotype of activated macrophages via EP2/EP4 receptors [8]. Although the specific roles of PGE_2_ and IL-10 were not examined by Geng and colleagues in their model of rhabdomyolysis-induced AKI, they convincingly demonstrated the active involvement of macrophage populations in mediating reno-protection by applying both depletion and reconstitution strategies at key time-points [1]. An important role for M2 macrophages in promoting renal parenchymal repair and regeneration has also been reported in other models of AKI, including unilateral ureteral obstruction, ischemia reperfusion, and adriamycin nephropathy [9–11]. Although multiple clinically relevant strategies have been identified in recent years for endogenous or exogenous re-programming of macrophages toward pro-repair phenotypes, this excellent study by Geng and colleagues strengthens the case that a single infusion of MSCs can potently expand M2 macrophage numbers *in vivo* at a critical time-point during the course of acute tissue injury.

Though not explicitly discussed by the authors, the fact that creatine kinase, a biomarker of muscle necrosis, was also significantly reduced in MSC-treated animals at all time-points suggests that rhabdomyolysis-associated AKI may be a particularly attractive therapeutic target for MSC infusion as a result of a ‘dual effect’ both on the severity of rhabdomyolysis itself and on the injured kidneys.

## Conclusions

As with many other diseases for which pre-clinical models indicate potential clinical benefit of MSCs in human subjects, the variances between certain findings of Geng and colleagues [1] and those reported by others [5, 6] in rhabdomyolysis-associated AKI highlight the need for further standardization within the field [12]. Conventional laboratory methods for isolating, expanding, and characterizing plastic-adherent MSCs inevitably result in heterogeneity of administered cells and ideally would be replaced by selective isolation of defined populations of cells from bone marrow or other tissues in order to pinpoint the precise distribution and mechanism(s) of action for a given dose and route of administration. Nonetheless, clinical trials of ‘off the shelf’ MSCs to protect against organ failure in acute rhabdomyolysis are theoretically quite feasible and, on the basis of the results of this well-performed study, may well have a high likelihood of success.

## Abbreviations

AKI: Acute kidney injury
IL: Interleukin
MSC: Mesenchymal stem (stromal) cell
PGE_2_: Prostaglandin E_2_.
